# *ranchSATdb*: A Genome-Wide Simple Sequence Repeat (SSR) Markers Database of Livestock Species for Mutant Germplasm Characterization and Improving Farm Animal Health

**DOI:** 10.3390/genes14071481

**Published:** 2023-07-20

**Authors:** Naveen Duhan, Simardeep Kaur, Rakesh Kaundal

**Affiliations:** 1 Department of Plants, Soils, and Climate/Center for Integrated BioSystems, College of Agriculture and Applied Sciences, Utah State University, Logan, UT 84322, USA; naveen.duhan@usu.edu (N.D.);; 2 Bioinformatics Facility, Center for Integrated BioSystems, Utah State University, Logan, UT 84322, USA; 3 Division of Biochemistry, ICAR-Indian Agricultural Research Institute, New Delhi 110012, India; 4 ICAR-Research Complex for North Eastern Hill Region (NEH), Umiam 793103, India

**Keywords:** microsatellite markers, ranch animals, livestock, SSRs, Germplasm characterization, population genetics

## Abstract

Microsatellites, also known as simple sequence repeats (SSRs), are polymorphic loci that play an important role in genome research, animal breeding, and disease control. Ranch animals are important components of agricultural landscape. The ranch animal SSR database, *ranchSATdb*, is a web resource which contains 15,520,263 putative SSR markers. This database provides a comprehensive tool for performing end-to-end marker selection, from SSRs prediction to generating marker primers and their cross-species feasibility, visualization of the resulting markers, and finding similarities between the genomic repeat sequences all in one place without the need to switch between other resources. The user-friendly online interface allows users to browse SSRs by genomic coordinates, repeat motif sequence, chromosome, motif type, motif frequency, and functional annotation. Users may enter their preferred flanking area around the repeat to retrieve the nucleotide sequence, they can investigate SSRs present in the genic or the genes between SSRs, they can generate custom primers, and they can also execute in silico validation of primers using electronic PCR. For customized sequences, an SSR prediction pipeline called *miSATminer* is also built. New species will be added to this website’s database on a regular basis throughout time. To improve animal health via genomic selection, we hope that *ranchSATdb* will be a useful tool for mapping quantitative trait loci (QTLs) and marker-assisted selection. The web-resource is freely accessible at https://bioinfo.usu.edu/ranchSATdb/.

## 1. Introduction

The United States has an impressive cattle and calf population of 89.3 million, demonstrating the importance of livestock in the country’s agricultural landscape [1]. Furthermore, the meat and poultry industry are the largest segment of US agriculture, accounting for 52 billion pounds of production in 2017. These staggering figures demonstrate livestock’s critical economic importance. Given the importance of the livestock industry to the economy, research efforts centered on selective breeding are critical. Selective breeding aims to improve desirable traits in livestock such as meat quality, milk production, disease resistance, and feed conversion efficiency. Several economically essential traits in chickens, cattle, sheep, horses, dogs, and pigs have been improved using traditional breeding programs and phenotypic selection, in which animals are directly selected based on estimated breeding values for each trait [2,3]. However, phenotypic selection has many drawbacks, including the fact that it can only be used for traits that are easily assessed, is more expensive, presents challenges in increasing disease resistance, and requires the raising of numerous individual animals because some traits are only visible in adults [4].

The study of an organism’s genetic makeup at the gene level is made possible by molecular genetics. Next-generation sequencing (NGS) technologies have transformed the field of genetics in the modern genomics era. NGS enables high-throughput DNA sequencing, allowing the analysis of thousands to millions of DNA fragments at the same time. This breakthrough has significantly accelerated the discovery and application of SSR markers in ranch animals [2]. The selection of economically important animal breeds through quantitative trait loci identification, linkage mapping, and association studies allows for more precise breeding strategies and faster genetic progress [5,6]. Additionally, using molecular genetics in breeding programs requires the ability to use DNA analysis to investigate organism genotypes for different mutations and PCR or non-PCR based markers [5,7,8,9,10]. One of the most crucial tools for genome analysis, population mapping, identifying potential disease-prevention candidates, and analyzing phylogenetic relationships between individuals is using molecular markers [11,12,13,14]. SSR markers have an edge due to high polymorphism, codominance, reproducibility, and transferability over other molecular markers. SSR analysis is simpler and less expensive than SNP genotyping, which frequently necessitates more advanced and expensive techniques like next-generation sequencing.

When compared to other molecular markers, microsatellites, also known as simple sequence repeats (SSR), are repetitions of one or more mono-, di-, tri-, tetra-, penta-, or hexa-nucleotide units that occur 1–6 times in tandem. SSRs have shown to be a powerful tool in investigations of genetic diversity, genetic mapping, and molecular breeding [15]—indeed, these have a high mutation rate and are widely distributed in eukaryotic genomes. Microsatellites have been used in population studies, diversity analysis, disease control, evolution analysis, and marker-assisted selection (MAS) to distinguish between different species or breeds [3,4]. The SSR discovery and functional analysis—demonstrating the importance of SSRs in complex characteristics and gene function—are being extensively used.

On a large scale, screening for simple sequence repeats (SSRs) in farm animal genomes using traditional methods such as genome libraries can be time-consuming and expensive. However, the development of in silico approaches for SSR prediction in recent years has revolutionized the process, allowing researchers to rapidly and practically generate molecular markers [16,17]. Taking advantage of this progress, the development of a user-friendly and comprehensive web resource containing readily available genetic information for farm animal species has proven invaluable. A resource like this can greatly aid research on farm species diversification, and other related topics. We present ‘*ranchSATdb*,’ a cutting-edge web resource designed to meet this need. *ranchSATdb* provides researchers with a variety of services, including SSR prediction, genotyping primer design, and ePCR-based polymorphism identification. This streamlines and simplifies the process of accessing and analyzing genetic information for farm animals by combining these features into a single platform. It is distinguished by its emphasis on SSR-based data tailored specifically for ranch animals. This database provides a comprehensive understanding of various molecular aspects of ranch animal genetics which includes a comprehensive set of tools and information for conducting research in this domain, ranging from SSR prediction to marker design. Researchers can gain valuable insights into the genetic makeup, diversity, and potential disease susceptibility of farm animal populations by leveraging the power of *ranchSATdb*. Finally, this resource helps to advance breeding strategies, disease control measures, and ranch animal species management and conservation.

## 2. Materials and Methods

### 2.1. Genomic Data Gathering

The genomic sequences of 12 farm animal species and available annotation information were downloaded. Among these, the genomes of 9 species (*Bos taurus*, *Capra hircus*, *Canis lupus familiaris*, *Felis catus*, *Equus caballus*, *Sus scrofa*, *Bos grunniens*, *Gallus gallus*, *Ovis* aries) were downloaded from ensembl (https://www.ensembl.org, accessed on 12 May 2022) and 3 species (*Bubalus bubalis*, *Apis melifera*, *Equus asinus*) genomes were retrieved from NCBI (https://www.ncbi.nlm.nih.gov, accessed on 12 May 2022). All the species genomes are assembled in chromosome level assembly. The gene feature file (GFF) containing information of genes in the assembly was also retrieved from the respected sources. All the genomes with their assembly version and GC content are presented in Table 1.

### 2.2. Computational Prediction of Simple Sequence Repeats and Functional Annotation

In this study, we used *miSATminer* [16,17], a customized Perl script we had designed for the prediction of simple sequence repeats (SSRs), to analyze these 12 genomes. We used pre-determined cutoff values based on previously published research to detect the presence of repeats. A minimum of 10 repeat units was required for mononucleotide repeats, while a minimum of 5 repeat units was required for dinucleotide, trinucleotide, tetranucleotide, pentanucleotide, and hexanucleotide repeats [16,17]. These thresholds were implemented during the SSR prediction process to ensure an accurate identification of the repeats within the genomes. SSR annotation was performed using our custom Python script called *SSRannotater*. *SSRannotater* was created to annotate predicted SSRs based on their location in various genomic regions. This script classified SSRs into exon, intron, and intergenic regions using gene feature files obtained from various species. To provide context for intronic repeats, the script assigned the closest gene. In the case of exons and intergenic repeats, the SSR was linked to the corresponding gene.

### 2.3. Database Development and Web Interface

**Figure 1:**
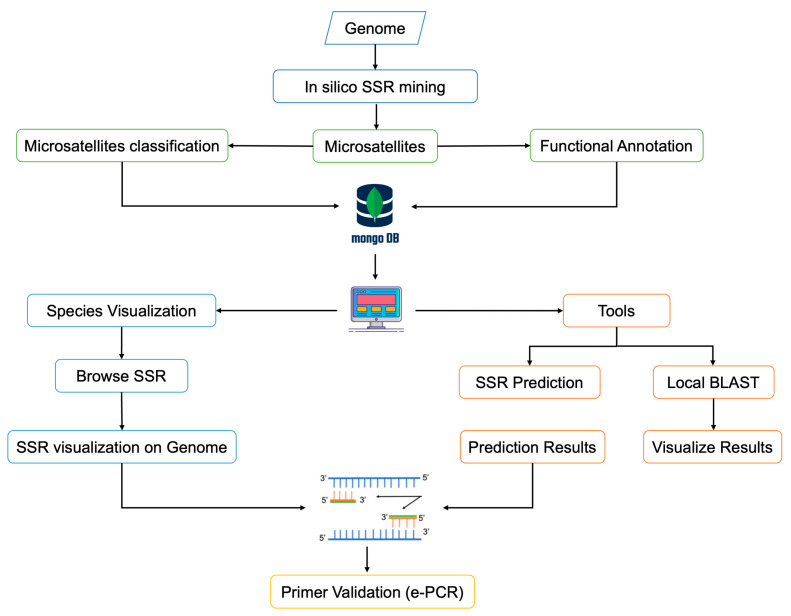
An overall workflow of *ranchSATdb*.

Ranch animals SSR database (*ranchSATdb*) is a three-tiered relational database webserver with a client-tier, middle-tier, and database-tier built using MERN stack technology and server via NodeJS. Predicted SSRs and their classification from all the 12 species were stored in a non-structure query language database MongoDB. The backend APIs were built with ExpressJS v4.17 and NodeJS v16, while the frontend was built with REACT. For novice users, the ‘Help page’ has also been made available for efficient database browsing. Furthermore, an information icon on the tools/features page provides brief information about the available functions and datasets. Several JavaScript chart libraries, including Morris, ChartJS, C3, Flot charts, and others, were used to implement real-time data visualization. Primer3 was implemented for real-time primer design with custom parameters. The *miSATminer* script was also implemented in the backend to predict SSRs for a user-specified query. For similarity searches and cross-species transferability, NCBI local BLAST and e-PCR were also implemented. The overall workflow of *ranchSATdb* is depicted in Figure 1.

## 3. Results and Discussion

### 3.1. Cross-Species Comparison of Ranch Animal Species SSRs

The *ranchSATdb* is a comprehensive web-resource for ranch animals represented by 12 species comprising 15,520,263 *in silico* predicted SSR markers. SSR markers have been shown to bind nuclear proteins and operate as transcriptional activators, and it is thought that they play a significant functional role in animal breeding programs [18]. The highest number of SSRs were found in *F. catus* while *A. melifera* had the fewest. The results are presented in Table 2. The number of predicted SSRs is directly proportional to genome size; the larger the genome, the more SSRs there are. SSR frequency (SSRs/MB) is frequently low in species with large genomes [19]. In our study of 12 ranch animal species, no correlation was found between genome size and SSR density. This is consistent with prior findings that there is no link between SSR density and genome size, and that changes in genome size may have an influence on the number of microsatellite repeats in the genome [14,20,21]. Because of their genetic codominance, abundance, hypervariability, genome dispersion, multiallelic variation, high repeatability, mendelian inheritance, and high degree of polymorphism, SSRs are employed for numerous applications in many animal species, e.g., cattle, pigs, sheep, dogs, and horses [12,15,22,23,24,25,26,27,28].

### 3.2. SSRs Characterization by Motif Type

**Figure 2:**
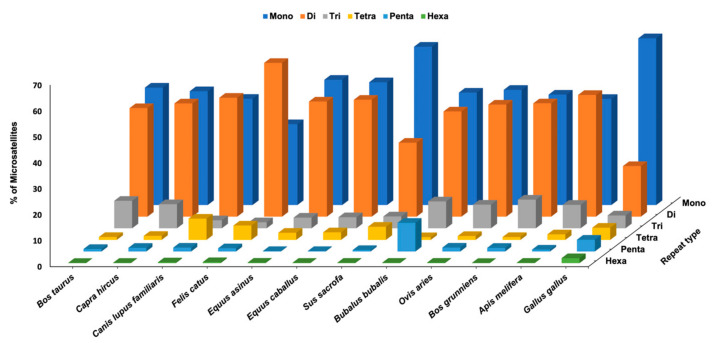
Distribution of SSRs based on motif type.

All the predicted SSRs loci in 12 ranch animal species were categorized into six groups: monomers, dimers, trimers, tetramers, pentamers, and hexamers. Approximately 85% of the SSRs in all species were monomers and dimers. *G. gallus* had the highest percentage for mono repeats, whereas *F. catus* had the lowest number of mono repeats; *B. grunniens*, *F. catus*, *C. lupus familiaris*, and *A. melifera* had more dimeric repeats than monomeric repeats (Figure 2). Almost all genomes have a significant abundance of monomeric repeats, which may be related to the inherent limits of next-generation sequencing (NGS) technologies used for data creation [29]. Similarly, dimeric repeats also recorded a higher abundance in animal genomes [27,30,31,32]. For trimeric repeat, *B. taurus* had the highest percentage and *F. catus* had the lowest percentage. In the case of tetrameric repeat, *C. lupus familiaris* had the highest percentage and *B. grunniens* had the lowest percentage. *B. bubalis* had the highest percentage (11.07%) of pentameric repeat, whereas *Equus caballus* and *E. asinus* had the lowest percentage (0.2%). For hexameric repeat, *G. gallus* had the highest percentage (1.92%) followed by *F. catus* (0.4%), whereas all other species has the lowest percentage (<0.3%). Longer repetitions were found to be less frequent in all SSR classes; other species have reported a decreased trend in SSR frequency with an increasing trend in repeat number [30,33,34].

### 3.3. Characterization of SSRs by Functional Annotation

**Figure 3:**
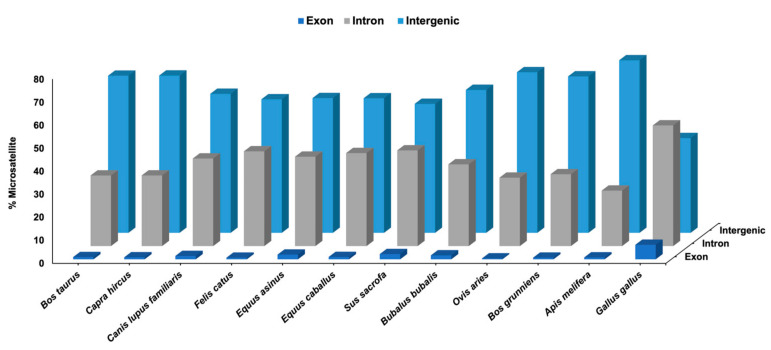
Distribution of SSRs based on genomic annotations.

**Figure 4:**
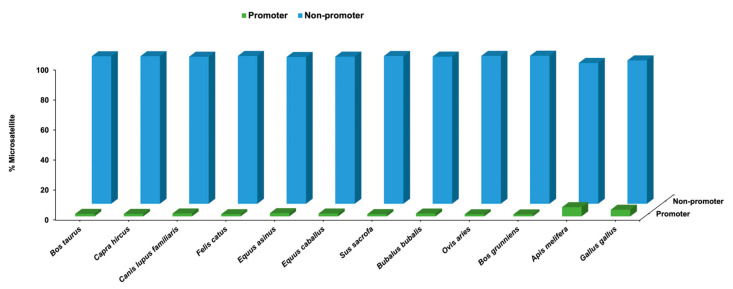
Distribution of SSRs based on promoter and non-promoter regions.

The predicted SSRs in each species were annotated with annotation files to classify exonic, intronic, and intergenic SSRs as well as promoter and non-promoter region SSRs. For the intronic SSRs, the closest gene was also added. This comprehensive annotation process enriched our understanding of the distribution and functional relevance of SSRs in different regions of the genome across multiple species. The highest number of exon region SSRs were found in *G. gallus*, whereas *O. aries* had the lowest number of SSRs. In case of intron regions, highest number of SSRs were annotated in *S. sacrofa*, and *O. aries* had the lowest number of SSRs. In the intergenic region, *O. aries* had the highest number of SSRs and *G. gallus* has the lowest percentage of SSRs as shown in Figure 3. In case of promoter region, highest percentage of SSRs were present in *A. melifera* and the lowest percentage were present in *B. grunniens*. Whereas, in the non-promoter region, the highest percentage of SSRs were present in *B. grunniens* and lowest number of SSRs were found in *A. melifera* as depicted in Figure 4.

### 3.4. Web Genomic Resource: ranchSATdb

A comprehensive database resource of microsatellite marker for ranch species is required because SSRs have numerous important applications in animal breeding. Even though species specific SSRs were reported in different species, there was no comprehensive database available that can offer information on several species with data on microsatellites, SSR predictions, e-PCR primer designs, visualization, etc. The ranch animals SSR database (*ranchSATdb*) web genomic resource was developed using a three-tier architecture. This is a comprehensive ranch animals SSR resource containing 12 species and 15,520,263 microsatellites. The web resource contains four major menus: Home, Species, Tools, and Help; some of the webpages have sub-pages. The ‘Home’ page provides the information about the web resource and general statistics of species available. The ‘Species’ page provides the information about the selected species. Predicted SSRs in each species can be filtered based on chromosome, coordinates on genome, repeat motif type, repeat motifs, minimum motif length, and annotation. The ‘Results’ page displays the real-time visualization of SSRs where users can select SSRs and visualize them on the genome by selecting up-stream and down-stream flank regions in range of 0 to 2000 nucleotides. The SSRs are highlighted in red on the selected region of the genome. The ‘Sequence’ page displays the motif features and is highlighted on the selected genomic region. Users can visualize the sequence and SSRs in table view or FASTA format view. Then the ‘design primer’ button can be used to access the design primer page where users can select custom parameters like GC content, Melting Temperature, primer size and product size to design primers. The primers page displays three set of primers and highlights them—in the genomic sequence as well. Using an e-PCR option, the chosen primers may be tested for cross-species transferability. The ‘Tools’ tab has two options: SSR prediction and BLAST. To predict SSR markers for user input sequences, *miSATminer* has been implemented on the backend, users may perform similarity searches on the BLAST search page. The ‘Help’ page offers a tutorial for properly utilizing the database as well as commonly asked questions. The overall *ranchSATdb* workflow is depicted in Figure 1. In future, tools like *Jbrowse* will be implemented to browse the SSRs with genic information on the genomes as well microsatellite markers from other ranch animal species will be added in the database.

### 3.5. Use of SSR Markers as an Efficient Tool

Our findings demonstrate the effectiveness of computational methods in accurate mining of the microsatellites. We successfully identified and extracted high-quality microsatellite sequences from genomic data using advanced algorithms and techniques. When compared to traditional approaches, these computational methods improved accuracy and speed, allowing for precise determination of repeat motifs, lengths, and locations. Cost and resource savings, as well as the ability to handle large-scale datasets efficiently, are all advantages of computational mining. This method has a lot of potential in population genetics, biodiversity research, genetic mapping, and breeding programs. Overall, our findings highlight the enormous value of computational methods in uncovering valuable genetic information and furthering our understanding of genetics and genomics [16,17,35]. Simple sequence repeats (SSRs) have proven to be useful in a variety of areas of genetic research. Individual identification is one prominent application, with SSRs used in DNA fingerprinting tests, pedigree and parentage analysis, and genome mapping. These markers are extremely useful for determining genetic relationships and tracing lineages within animal populations. SSRs are also important in studying phylogenetic relationships and the genetic structure of animal populations. SSRs enable the development of diversity measures that aid in rating breeds for conservation efforts by assessing genetic diversity within and across breeds. This information is critical for developing effective breeding strategies and preserving genetic diversity in livestock species [36]. Furthermore, SSRs aid in the identification of disease-associated quantitative trait loci (QTLs), allowing for a better understanding of genetic factors influencing disease susceptibility in ranch animals. This understanding improves disease control measures and enables targeted breeding programs for disease resistance. Recognizing the importance of SSRs in ranch animals, we created a SSR-based database. This database might serve as an invaluable resource for genomic research and disease management in these animals. The database facilitates efficient and accurate genetic analysis by providing comprehensive SSR information and associated tools, assisting researchers in unraveling the molecular aspects of ranch animal genetics. Finally, the use of SSR-based databases and tools improves our understanding of ranch animals’ genetic makeup, interbreed relationships, and relevance in conservation breeding efforts. This knowledge helps to manage and preserve diverse ranch animal populations, allowing for more sustainable agricultural practices and improved disease control measures.

According to few studies [37,38], SSRs can be used for MAS in breeding practices and for mapping QTLs for functional and production traits in animals. Moreover, SSRs are also necessary for locating the functional and positional candidate genes underlying quantitative traits. Therefore, research into the potential biological function and evolutionary significance of SSRs is helping to better understand the animal genomics. The existing set of markers holds significant potential for enhancing traits and species characterization. SSR markers have also been successfully used to identify various diseases and to detect mutations in ranch animals with genetic disorders, for instance, disease resistance quantitative trait loci, such as trypan tolerance in cattle, nematode resistance in sheep, and *E. coli* resistance in pigs [39,40,41]. In Qinghai Bamei pigs, which are valuable DNA markers in animal breeding identified three novel candidate SSRs loci, namely (ATC)n-P1, (AC)n-P2, and (AC)n-P3, were examined using Time-of-flight mass spectrometry (TOF-MS) genotyping. Further, they investigated the relationship between these SSRs and litter size in Qinghai Bamei sows using association analyses. The genotyping results reported varying numbers of genotypes and alleles at each locus, with most loci exhibiting high polymorphism information content (PIC) values. The (ATC)n-P1 locus was found to have a significant association with litter size, implying that it could be used as a marker for marker-assisted selection (MAS) in pig breeding [22]. In another study on four miniature pig breeds (Wuzhishan, Bama, Luchuan, and Zangxiang), SSRs were identified and characterized using sequencing data from SSR-enriched libraries to understand their genome-wide characteristics and polymorphisms. They found variation in types, number, and distribution of SSRs in all four breeds. Further, they compared theses SSRs against Duroc pig reference genome to find unique and common polymorphic SSRs associated with genes involved in growth and development, such as FGF23, MYF6, IGF1R and LEPROT [34]. A total of 20 SSR markers genotyping 17 Turkish water buffalo populations reported deviation from Hardy–Weinberg equilibrium due to non-random breeding. The overall polymorphic information content of the microsatellite loci indicated their suitability for genetic diversity analysis [42]. Ref. [3] reported that in the case of wild bighorn sheep, candidate gene SSR variation in locus of TCRG4 is linked with parasitism. The availability of such markers enables the application of high-density linkage mapping, facilitating the discovery of genes associated with specific traits. This molecular approach paves the way for targeted trait improvement, as it allows for a focused exploration of the underlying genetic factors.

### 3.6. Applications and Features of Database

The *ranchSATdb* is a valuable resource that can be used for animal breeding, conservation, and genetic research. The database is especially useful for studying the genetic diversity and structure of ranch animal populations, allowing researchers to gain insight into the effects of various factors on these populations. Researchers can assess the impact of breeding practices, habitat fragmentation, and population bottlenecks on the genetic makeup of ranch animals by analyzing SSR markers available in the *ranchSATdb*. This data is essential for developing effective conservation strategies and managing genetic variation within populations. The *ranchSATdb*’s advanced search options, which allow users to explore the database based on parameters such as marker frequency, motif type, repeat type, and chromosome range, is one of its most important features. Researchers can now tailor their searches to specific genetic traits or regions of interest, allowing for more focused investigations into the genetic diversity of ranch animal populations. The database’s ability to visualize SSRs in real time is particularly useful. By selecting upstream and downstream flank regions, researchers can select specific SSR markers and visualize their locations on the genome. This visual representation aids in the identification of regions of interest for further genetic studies by improving understanding of the distribution and clustering of SSRs across the genome. Furthermore, the *ranchSATdb* supports primer design, which is required for SSR marker experimental validation. Users can tailor primer parameters such as GC content, melting temperature, primer size, and product size to meet their specific research requirements. This feature facilitates downstream applications such as genotyping and parentage verification by streamlining the process of designing primers for PCR amplification. The database also includes SSR prediction and BLAST searches. *miSATminer*, a powerful algorithm previously published by us, is used by the SSR prediction tool to predict SSR markers based on user-supplied sequences. This functionality enables researchers to discover new SSR markers in their own sequences of interest, increasing the number of markers available for genetic studies. Users can use the BLAST search tool to perform similarity searches using the BLAST algorithm, which facilitates comparative analysis and identifies potential homologous regions across different species. The *ranchSATdb* can serve as a valuable resource for ranch animal breeding programs that require parentage verification and pedigree reconstruction. SSR markers are ideal for determining the parentage of offspring due to their high polymorphism. Researchers can accurately establish parentage and reconstruct pedigrees by comparing the genotypes of potential parents with those of the offspring. These data are critical for keeping breeding records, optimizing breeding strategies, and ensuring the accuracy of genetic evaluations in ranch animal populations.

## 4. Conclusions

*ranchSATdb*, an online resource for ranch animals SSR analysis, has been developed with advanced GUI capabilities, providing comprehensive marker selection services for farm animal species. The database contains a vast collection of 15,520,263 microsatellites from 12 ranch animals. These markers offer cross-species transferability, addressing the need for molecular markers in ranch animal species lacking whole-genome sequencing data. The global research community can benefit significantly from this genetic online resource. The *ranchSATdb* offers a range of unique features, enabling users to search, predict, analyze, and visualize SSRs. Users can design custom primers based on desired amplicon size and perform in silico validation with ePCR. Real-time graphical visualization of predicted SSRs, exploration of SSR sequences by modifying the flanking region, and identification of related genes with functional annotation information are additional functionalities. The user-friendly interface also allows users to input a nucleotide sequence of interest, adjust parameters, and design primers using the online server. With the high-performance cluster computing, *ranchSATdb* ensures fast and accurate prediction of SSRs. This enables mining of chromosome-wise microsatellite loci, primer design for genic and non-genic FDM-SSRs (functional domain markers), and efficient identification of polymorphisms through e-PCR, essential for future re-sequencing efforts. *ranchSATdb* serves multiple purposes, including marker-assisted breeding improvement in ranch animals, genetic linkage mapping, QTL identification, and other knowledge-seeking research endeavors. The web resource will continue to expand its coverage to include more ranch animal species in the future. Interested users can freely access the web resource at https://bioinfo.usu.edu/ranchSATdb/, accessed on 10 July 2023.

## Figures and Tables

**Table 1:** Genome information.

Genome Name	Genome Assembly	GC Content (%)
*B. taurus*	ARS-UCD1.3	41.5
*C. hircus*	ARS1.2	43
*C. lupus familiaris*	Dog10K_Boxer_Tasha	41
*F. catus*	F.catus_Fca126_mat1.0	41.5
*E. asinus*	ASM1607732v2	41
*E. caballus*	EquCab3.0	41
*S. sacrofa*	Sscrofa11.1	41.5
*B. bubalis*	NDDB_SH_1	41.5
*O. aries*	ARS-UI_Ramb_v2.0	41.5
*B. grunniens*	BosGru3.1	41.5
*A. melifera*	Amel_HAv3.1	32.5
*G. gallus*	bGalGal1.mat.broiler.GRCg7b	42

**Table 2:** Statistics of species-wise microsatellites based on genome size, number of base-pairs, number of SSRs per mega-base-pairs, genome coverage.

Species Name	Size in MB	No. of SSR	Freq/Mb	Genome Coverage (%)
*B. taurus*	2628.39	1,176,611	447.65	16.62
*C. hircus*	2466.19	1,064,647	431.70	15.22
*C. lupus familiaris*	2361.80	2,364,725	1001.23	39.44
*F. catus*	2460.25	2,868,494	1165.94	46.81
*E. asinus*	2431.35	892,223	366.97	11.98
*E. caballus*	2409.14	862,486	358.01	11.54
*S. sacrofa*	2435.26	1,789,672	734.90	28.14
*B. bubalis*	2622.44	1,204,611	459.35	17.19
*O. aries*	2809.02	1,230,663	438.11	17.93
*B. grunniens*	2823.19	1,252,028	443.48	18.44
*A. melifera*	222.84	310,585	1393.76	4.65
*G. gallus*	1061.13	503,518	474.51	7.99

## Data Availability

All data generated in this study are included in the database. The database is freely available at https://bioinfo.usu.edu/ranchSATdb/, accessed on 10 July 2023.

## References

[1] Katti M.V., Ranjekar P.K., Gupta V.S. (2001). Differential Distribution of Simple Sequence Repeats in Eukaryotic Genome Sequences. Mol. Biol. Evol..

[2] Chombe D., Bekele E. (2018). Genetic Diversity Analysis of Cultivated Korarima [Aframomum Corrorima (Braun) P.C.M. Jansen] Populations from Southwestern Ethiopia Using Inter Simple Sequence Repeats (ISSR) Marker. J. Biol. Res.-Thessalon..

[3] Xiao Y., Xia W., Ma J., Mason A.S., Fan H., Shi P., Lei X., Ma Z., Peng M. (2016). Genome-Wide Identification and Transferability of Microsatellite Markers between Palmae Species. Front. Plant Sci..

[4] Abd El-Hack M.E., Abdelnour S.A., Swelum A.A., Arif M. (2018). The Application of Gene Marker-Assisted Selection and Proteomics for the Best Meat Quality Criteria and Body Measurements in Qinchuan Cattle Breed. Mol. Biol. Rep..

[5] Coltman D.W., Wilson K., Pilkington J.G., Stear M.J., Pemberton J.M. (2001). A Microsatellite Polymorphism in the γ Interferon Gene Is Associated with Resistance to Gastrointestinal Nematodes in a Naturally-Parasitized Population of Soay Sheep. Parasitology.

[6] Hanotte O., Ronin Y., Agaba M., Nilsson P., Gelhaus A., Horstmann R., Sugimoto Y., Kemp S., Gibson J., Korol A. (2003). Mapping of Quantitative Trait Loci Controlling Trypanotolerance in a Cross of Tolerant West African N’Dama and Susceptible East African Boran Cattle. Proc. Natl. Acad. Sci. USA.

[7] Ünal E.Ö., Işık R., Şen A., Kuş E.G., Soysal M.İ. (2021). Evaluation of Genetic Diversity and Structure of Turkish Water Buffalo Population by Using 20 Microsatellite Markers. Animals.

[8] United States Cattle Inventory Down 3%. https://www.nass.usda.gov/Newsroom/2023/01-31-2023.phpwww.nass.usda.gov/Newsroom/2023/01-31-2023.php.

[9] Reshma R.S., Das D.N. (2021). Molecular Markers and Its Application in Animal Breeding. Advances in Animal Genomics.

[10] Luikart G., Pilgrim K., Visty J., Ezenwa V.O., Schwartz M.K. (2008). Candidate Gene Microsatellite Variation Is Associated with Parasitism in Wild Bighorn Sheep. Biol. Lett..

[11] Teneva A., Dimitrov K., Petrović C.V., Petrović M.P., Dimitrova I., Tyufekchiev N., Petrov N. (2013). Molecular Genetics and SSR Markers as a New Practice in Farm Animal Genomic Analysis for Breeding and Control of Disease Disorders. Biotechnol. Anim. Husb..

[12] Bishop S.C., Woolliams J.A. (2014). Genomics and Disease Resistance Studies in Livestock. Livest. Sci..

[13] Hu G., Do D.N., Gray J., Miar Y. (2020). Selection for Favorable Health Traits: A Potential Approach to Cope with Diseases in Farm Animals. Animals.

[14] Hospital F., Goldringer I., Openshaw S. (2000). Efficient Marker-Based Recurrent Selection for Multiple Quantitative Trait Loci. Genet. Res..

[15] Andersson L. (2001). Genetic Dissection of Phenotypic Diversity in Farm Animals. Nat. Rev. Genet..

[16] Compton S.R. (2020). PCR and RT-PCR in the Diagnosis of Laboratory Animal Infections and in Health Monitoring. J. Am. Assoc. Lab. Anim. Sci..

[17] Dekkers J.C.M., Hospital F. (2002). The Use of Molecular Genetics in the Improvement of Agricultural Populations. Nat. Rev. Genet..

[18] Charon K.M., Moskwa B., Rutkowski R., Gruszczyńska J., Świderek W. (2002). Microsatellite Polymorphism in DRB1 Gene (MHC Class II) and Its Relation to Nematode Faecal Egg Count in Polish Heath Sheep. J. Anim. Feed. Sci..

[19] Adamov N., Mickov L., Petkov V., Adamov M. (2011). Microsatellite Markers for Pedigree Verification in Cattle. Maced. J. Anim. Sci..

[20] Svishcheva G., Babayan O., Lkhasaranov B., Tsendsuren A., Abdurasulov A., Stolpovsky Y. (2020). Microsatellite Diversity and Phylogenetic Relationships among East Eurasian Bos Taurus Breeds with an Emphasis on Rare and Ancient Local Cattle. Animals.

[21] Zhao X., Tian Y., Yang R., Feng H., Ouyang Q., Tian Y., Tan Z., Li M., Niu Y., Jiang J. (2012). Coevolution between Simple Sequence Repeats (SSRs) and Virus Genome Size. BMC Genom..

[22] Zhou Q., Luo D., Ma L., Xie W., Wang Y., Wang Y., Liu Z. (2016). Development and Cross-Species Transferability of EST-SSR Markers in Siberian Wildrye (*Elymus sibiricus* L.) Using Illumina Sequencing. Sci. Rep..

[23] Duhan N., Meshram M., Loaiza C.D., Kaundal R. (2020). CitSATdb: Genome-Wide Simple Sequence Repeat (SSR) Marker Database of Citrus Species for Germplasm Characterization and Crop Improvement. Genes.

[24] Duhan N., Kaundal R. (2021). Legumessrdb: A Comprehensive Microsatellite Marker Database of Legumes for Germplasm Characterization and Crop Improvement. Int. J. Mol. Sci..

[25] Salisu I.B., Olawale A.S., Jabbar B., Koloko B.L., Abdurrahaman S.L., Amin A.B., Ali Q. (2018). Molecular Markers and Their Potentials in Animal Breeding and Genetics. Niger. J. Anim. Sci..

[26] Morgante M., Hanafey M., Powell W. (2002). Microsatellites Are Preferentially Associated with Nonrepetitive DNA in Plant Genomes. Nat. Genet..

[27] Portis E., Lanteri S., Barchi L., Portis F., Valente L., Toppino L., Rotino G.L., Acquadro A. (2018). Comprehensive Characterization of Simple Sequence Repeats in Eggplant (*Solanum melongena* L.) Genome and Construction of a Web Resource. Front. Plant Sci..

[28] Portis E., Portis F., Valente L., Moglia A., Barchi L., Lanteri S., Acquadro A. (2016). A Genome-Wide Survey of the Microsatellite Content of the Globe Artichoke Genome and the Development of a Web-Based Database. PLoS ONE.

[29] Wu G., Shen W., Xue X., Wang L., Ma Y., Zhou J. (2021). A Novel (ATC)n Microsatellite Locus Is Associated with Litter Size in an Indigenous Chinese Pig. Vet. Med. Sci..

[30] Yu H., Zhao Y., Iqbal A., Xia L., Bai Z., Sun H., Fang X., Yang R., Zhao Z. (2021). Effects of Polymorphism of the GPAM Gene on Milk Quality Traits and Its Relation to Triglyceride Metabolism in Bovine Mammary Epithelial Cells of Dairy Cattle. Arch. Anim. Breed..

[31] Beuzen N.D., Stear M.J., Chang K.C. (2000). Molecular Markers and Their Use in Animal Breeding. Vet. J..

[32] Xu P., Lu C., Sun Z., Kuang Y., Cao D., Huo T., Li C., Jin H., Zheng X. (2022). In Silico Screening and Development of Microsatellite Markers for Genetic Analysis in Perca Fluviatilis. Animals.

[33] Yang W., Bai Z., Wang F., Zou M., Wang X., Xie J., Zhang F. (2022). Analysis of the Genetic Diversity and Population Structure of Monochasma Savatieri Franch. Ex Maxim Using Novel EST-SSR Markers. BMC Genom..

[34] Tóth G., Gáspári Z., Jurka J. (2000). Microsatellites in Different Eukaryotic Genomes: Survey and Analysis. Genome Res..

[35] Tautz D., Renz M. (1984). Simple Sequences Are Ubiquitous Repetitive Components of Eukaryotic Genomes. Nucleic Acids Res..

[36] Haseneyer G., Schmutzer T., Seidel M., Zhou R., Mascher M., Schön C.C., Taudien S., Scholz U., Stein N., Mayer K.F.X. (2011). From RNA-Seq to Large-Scale Genotyping—Genomics Resources for Rye (*Secale cereale* L.). BMC Plant Biol..

[37] Srivastava S., Avvaru A.K., Sowpati D.T., Mishra R.K. (2019). Patterns of Microsatellite Distribution across Eukaryotic Genomes. BMC Genom..

[38] Subramanian S., Mishra R.K., Singh L. (2003). Genome-Wide Analysis of Microsatellite Repeats in Humans: Their Abundance and Density in Specific Genomic Regions. Genome Biol..

[39] Wang H., Fu Y., Gu P., Zhang Y., Tu W., Chao Z., Wu H., Cao J., Zhou X., Liu B. (2020). Genome-Wide Characterization and Comparative Analyses of Simple Sequence Repeats among Four Miniature Pig Breeds. Animals.

[40] Groeneveld L.F., Lenstra J.A., Eding H., Toro M.A., Scherf B., Pilling D., Negrini R., Finlay E.K., Jianlin H., Groeneveld E. (2010). Genetic Diversity in Farm Animals—A Review. Anim. Genet..

[41] Kriangwanich W., Nganvongpanit K., Buddhachat K., Siengdee P., Chomdej S., Ponsuksili S., Thitaram C. (2020). Genetic Variations and Dog Breed Identification Using Inter-Simple Sequence Repeat Markers Coupled with High Resolution Melting Analysis. PeerJ.

[42] Meijerink E., Neuenschwander S., Fries R., Dinter A., Bertschinger H.U., Stranzinger G., Vögeli P. (2000). A DNA Polymorphism Influencing α(1,2)Fucosyltransferase Activity of the Pig FUT1 Enzyme Determines Susceptibility of Small Intestinal Epithelium to *Escherichia coli* F18 Adhesion. Immunogenetics.

